# Identification of *Klebsiella pneumoniae, Klebsiella quasipneumoniae, Klebsiella variicola* and Related Phylogroups by MALDI-TOF Mass Spectrometry

**DOI:** 10.3389/fmicb.2018.03000

**Published:** 2018-12-07

**Authors:** Carla Rodrigues, Virginie Passet, Andriniaina Rakotondrasoa, Sylvain Brisse

**Affiliations:** ^1^Biodiversity and Epidemiology of Bacterial Pathogens, Institut Pasteur, Paris, France; ^2^Institut Pasteur Madagascar, Madagascar, Africa

**Keywords:** *Klebsiella pneumoniae*, phylogroups, MALDI-TOF MS, identification, biomarkers

## Abstract

*Klebsiella pneumoniae* (phylogroup Kp1), one of the most problematic pathogens associated with antibiotic resistance worldwide, is phylogenetically closely related to *K. quasipneumoniae* [subsp. *quasipneumoniae* (Kp2) and subsp. *similipneumoniae* (Kp4)], *K. variicola* (Kp3) and two unnamed phylogroups (Kp5 and Kp6). Together, Kp1 to Kp6 make-up the *K. pneumoniae* complex. Currently, the phylogroups can be reliably identified only based on gene (or genome) sequencing. Misidentification using standard laboratory methods is common and consequently, the clinical significance of *K. pneumoniae* complex members is imprecisely defined. Here, we evaluated and validated the potential of MALDI-TOF mass spectrometry (MS) to discriminate *K. pneumoniae* complex members. We detected mass spectrometry biomarkers associated with the phylogroups, with a sensitivity and specificity ranging between 80–100% and 97–100%, respectively. Strains within phylogroups Kp1, Kp2, Kp4, and Kp5 each shared two specific peaks not observed in other phylogroups. Kp3 strains shared a peak that was only observed otherwise in Kp5. Finally, Kp6 had a diagnostic peak shared only with Kp1. Kp3 and Kp6 could therefore be identified by exclusion criteria (lacking Kp5 and Kp1-specific peaks, respectively). Further, ranked Pearson correlation clustering of spectra grouped strains according to their phylogroup. The model was tested and successfully validated using different culture media. These results demonstrate the potential of MALDI-TOF MS for precise identification of *K. pneumoniae* complex members. Incorporation of spectra of all *K. pneumoniae* complex members into reference MALDI-TOF spectra databases, in which they are currently lacking, is desirable. MALDI-TOF MS may thereby enable a better understanding of the epidemiology, ecology, and pathogenesis of members of the *K. pneumoniae* complex.

## Introduction

*Klebsiella pneumoniae* is an increasingly challenging human bacterial pathogen, causing hospital or community-acquired infections that are associated with high rates of antibiotic resistance (Wyres and Holt, 2016; European Centre for Disease Prevention and Control [ECDC], 2017). Population diversity studies have shown that *K. pneumoniae* is phylogenetically closely related to *K. quasipneumoniae* (subsp. *quasipneumoniae* and subsp. *similipneumoniae*) and *K. variicola* (Brisse and Verhoef, 2001; Holt et al., 2015; Blin et al., 2017). Before recent taxonomic updates (Rosenblueth et al., 2004; Brisse et al., 2014), *K. pneumoniae* and the other above taxa were designed as *K. pneumoniae* phylogroups Kp1, Kp2, Kp4, and Kp3, respectively (Brisse et al., 2004). Together with two novel phylogroups (Kp5 and Kp6) that were recently described (Blin et al., 2017), these taxa constitute the *K. pneumoniae* complex. Note that Kp6 corresponds to a phylogroup proposed to be named as *K. quasivariicola* (Long et al., 2017a). *K. pneumoniae* (*sensu stricto*) is the major cause of human and animal infections within the complex. However, the involvement in human infections of the other members of the complex is gaining recognition (Brisse et al., 2004; Seki et al., 2013; Maatallah et al., 2014; Holt et al., 2015; Breurec et al., 2016; Becker et al., 2018). Unfortunately, the unsuitability of traditional clinical microbiology methods to distinguish species within the complex leads to high rates of misidentifications (most often as *K. pneumoniae*) that are masking the true clinical significance of each phylogroup and their potential epidemiological specificities (Brisse et al., 2004; Seki et al., 2013; Long et al., 2017b; Becker et al., 2018). In fact, the different members of the *K. pneumoniae* complex can only be reliably identified based on whole-genome sequencing (WGS) or sequencing of specific genetic markers (e.g., *bla*_LEN_, *bla*_OKP_, *bla*_SHV_,*rpoB, gyrA, parC*) (Haeggman et al., 2004; Brisse et al., 2014; Holt et al., 2015). However, the latter methods are not available for most of the routine laboratories and are limited in speed, cost and throughput. In the last years, some PCR-based identification methods were developed but they are prone to errors or do not distinguish all phylogroups (Brisse et al., 2004; Bialek-Davenet et al., 2014b; Garza-Ramos et al., 2015; Fonseca et al., 2017). Clearly, there is a need for a reliable, cost-effective and fast identification method able to discriminate members of the *K. pneumoniae* complex.

Matrix-assisted laser desorption ionization-time of flight (MALDI-TOF) mass spectrometry (MS) has revolutionized routine identification of microorganisms, being a fast and cost-effective technique. It now represents a first line identification method in many clinical, environmental, and food microbiology laboratories (van Belkum et al., 2017). In the case of the *K. pneumoniae* complex, MALDI-TOF MS identification remains largely unsatisfactory given the absence of well-characterized, representative members of the complex in spectral databases. Currently, only *K. pneumoniae* and *K. variicola* are included in the Bruker database ^1^, and identification of even these two species is imprecise given the lack of reference spectra of other phylogroups (Berry et al., 2015; Long et al., 2017b; Dinkelacker et al., 2018). To address this important limitation of currently MALDI-TOF MS technology, we used a collection of well-characterized strains from the six *K. pneumoniae* complex phylogroups and analyzed them by MALDI-TOF MS in order to define the potential of this method to identify species within the *K. pneumoniae* complex (Rodrigues et al., 2018). In addition, we validated our MALDI-TOF MS based model using a test collection of 49 isolates belonging to the *K. pneumoniae* complex, with spectra obtained from different culture media and extraction procedures.

## Materials and Methods

### Bacterial Strains

A set of 46 strains previously identified by WGS or using core gene sequences (Bialek-Davenet et al., 2014a; Brisse et al., 2014; Blin et al., 2017; Passet and Brisse, 2018) were analyzed in this study (Supplementary Table S1). The strains belonged to the taxa *K. pneumoniae* (*sensu stricto*, i.e., Kp1; *n* = 10), *K. quasipneumoniae* subsp. *quasipneumoniae* (Kp2, *n* = 9), *K. quasipneumoniae* subsp. *similipneumoniae* (Kp4, *n* = 7), *K. variicola* (Kp3, *n* = 9), and to two taxonomically undefined lineages named Kp5 (*n* = 6) and ‘*K. quasivariicola*’ (Kp6, *n* = 5). The strains represented a diversity of multilocus sequence typing (MLST) types within each phylogroup. Strains had been stored in brain heart infusion broth containing 25% glycerol at -80°C and were sub-cultivated before use in this study.

### Spectra Acquisition

An overnight culture on Luria-Bertani agar (37°C, 18 h) was used to prepare the samples with the ethanol/formic acid extraction procedure following the manufacturer recommendations (Bruker Daltonics, Bremen, Germany). Samples (1 μL) were spotted onto an MBT Biotarget 96 target plate, air dried and overlaid with 1 μL of a saturated α-cyano-4-hydroxycinnamic acid (HCCA) matrix solution in 50% of acetonitrile and 2.5% of trifluoroacetic acid. Mass spectra were acquired on a Microflex LT mass spectrometer (Bruker Daltonics, Bremen, Germany) using the default parameters (detection in linear positive mode, laser frequency of 60 Hz, ion source voltages of 2.0 and 1.8 kV, lens voltage of 6 kV) within the *m/z* of 2,000–20,000. For each strain, a total of 24 spectra from eight independent spots were acquired (three spectra *per* spot, instrumental replicates, one single day) according to the main spectra protocol (MSP). External calibration of the mass spectra was performed using Bruker Bacterial Test Standard (BTS).

### Spectra Analysis

The spectra were preprocessed by applying the “smoothing” and “baseline subtraction” procedures available in FlexAnalysis software (Bruker Daltonics, Bremen, Germany), exported as peak lists with *m/z* values and signal intensities for each peak in text format, and imported into a dedicated BioNumerics v7.6 (Applied Maths, Ghent, Belgium) database. Peak detection was performed in BioNumerics using a signal to noise ratio of 20. The instrumental replicates (24 spectra for each strain) were used to generate a mean spectrum for each strain using the following parameters: minimum similarity, 90%; minimum peak detection rate, 60%; constant tolerance, 1; and linear tolerance, 300 ppm. Finally, peak matching was performed to search all distinct peaks (called peak classes in BioNumerics) using as parameters: constant tolerance, 1.9; linear tolerance, 550 ppm; maximum horizontal shift, 1; peak detection rate, 10. The discriminating value of each resulting peak was evaluated by a Mann–Whitney test (Vranckx et al., 2017). In order to test and validate our results an identification project was constructed in a BioNumerics database, using our spectra as reference set and a support vector machine (SVM, supervised algorithm) as classifier (cross-validation procedure). The application of SVMs classifier algorithms is very useful to discriminate between groups when the differences are minimal (DeMarco and Ford, 2013). In the cross-validation procedure, 70% of the available data (randomly selected) were used as model, whereas the 30% remaining spectra were used as test in order to assess the proportion (%) of correct predictions for each phylogroup. To allocate proteins associated with the specific peaks, the online tool TagIdent was used^2^. In fact, this tool allows the identification of proteins by their mass considering all the proteins available in UniProt Knowledgebase (Swiss-Prot and TrEMBL) for the taxonomic group under study. Additionally, a Neighbor Joining tree based on ranked Pearson coefficient was constructed using BioNumerics.

### External Validation Dataset

Forty-nine isolates belonging to *K. pneumoniae* phylogroups Kp1 (*n* = 23), Kp2 (*n* = 7), Kp3 (*n* = 7), Kp4 (*n* = 9) and Kp5 (*n* = 3), previously characterized by WGS were used to assess the robustness of the MALDI-TOF MS method (no Kp6 isolates other than those used in the model construction were available). These isolates are part of a study collection recovered from fecal samples of healthy carriers in Madagascar (2015–2016) (under the BioProject PRJEB29143). The Kp1 test isolates included producers of ESBL or AmpC enzymes and represented a snapshot of clinically relevant multidrug resistant sublineages (2 isolates of ST17, 2 ST48, 2 ST101, 1 ST14, 1 ST20, 1 ST25, 1 ST45, 1 ST307, 1 ST375, 1 ST380). Isolates from Kp3 and Kp4 were randomly selected, and all available isolates of Kp2 and Kp5 were included. In order to evaluate the impact of different culture media and different extraction procedures, spectra were acquired in triplicate using four different experimental conditions: bacteria were grown overnight on Luria-Bertani agar (37°C, 18 h) and Columbia agar plus 5% sheep blood (37°C, 18 h), and from each culture, were either directly transferred onto MALDI targets, or were cell extracted (using the ethanol/formic acid extraction procedure). Obtained MALDI-TOF spectra were then projected in our model using the identification project previously constructed.

## Results and Discussion

Forty-six strains representing a diversity of genotypes within the six phylogroups currently known within the *K. pneumoniae* complex (Supplementary Table S1) were analyzed by MALDI-TOF MS. Based on the MALDI Biotyper Compass database version 4.1.80 (Bruker Daltonics, Bremen, Germany), the 46 strains were identified either as *K. pneumoniae* (31 strains, all belonging to Kp1, Kp2, Kp4, and Kp6) or as *K. variicola* (15 strains, all strains of Kp3 and Kp5). Identification scores ranged between 2.16–2.56 for *K. pneumoniae* and 1.89–2.55 for *K variicola.* Of note, in two cases (Kp1-SB1139 and Kp6-SB6071) a replicate was reported in one measure as *K. pneumoniae* and in other as *K. variicola*. These data highlight the need to update the database in order to refine confidence in *K. pneumoniae*/*K. variicola* identification and to enable identification of *K. quasipneumoniae* and novel phylogroups.

**FIGURE 1:**
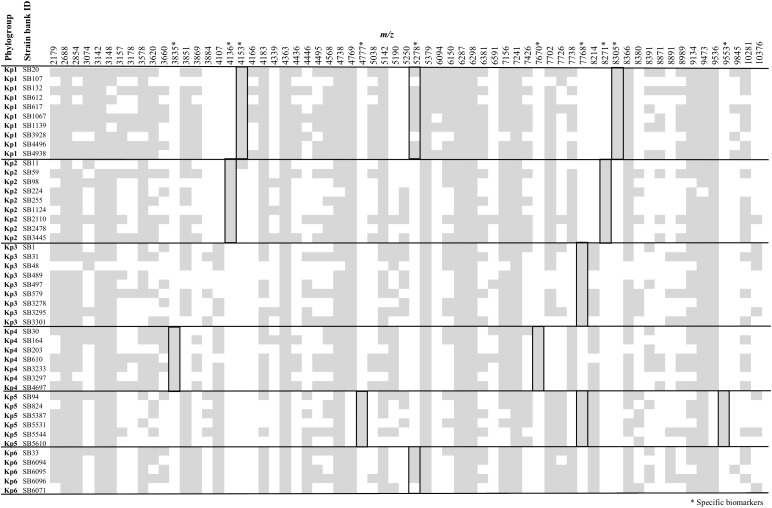
Peak positions (*m/z*) for each of the *Klebsiella pneumoniae* complex strains. Stars denote those peaks that are useful for discrimination among phylogroups, as detailed in Table 1.

Figure 1 summarizes the peak positions found in each strain. Most (about 97%) of the peaks were concentrated in the region below 10,000 *m/z* and almost no peak was found above this value. The similarity among spectra within the *K. pneumoniae* complex was always above 87% (data not shown), with peaks at 4363, 5379, 6287, 6298, 7241, and 9473 *m/z* being found in all the members of the complex. Importantly, 10 specific biomarkers associated with specific members of the *K. pneumoniae* complex were identified. These peaks were located within the range 3835–9553 *m/z*. Based on the current dataset, the specificity and sensitivity of their distribution among phylogroups ranged between 97–100 and 80–100%, respectively (Figure 1 and Table 1). Kp1 (4153 and 8305 *m/z*), Kp2 (4136 and 8271 *m/z*), Kp4 (7670 and 3835 *m/z*), and Kp5 (4777 and 9553 *m/z*) each presented two specific peaks, which may allow their unambiguous identification. Interestingly, all the pair peaks detected (Kp1, Kp2, Kp4, and Kp5) always exhibited approximately half of the *m/z* ratio of the other peak, which might correspond to the single and double charged protein ions, as often observed in MALDI-TOF MS experiments (Fagerquist, 2017). Kp3 strains shared a peak that was only observed otherwise in Kp5 (7768 *m/z*). Finally, Kp6 had a diagnostic peak (5278 *m/z*) shared only with Kp1. Kp3 and Kp6 could therefore be identified by exclusion criteria (lacking Kp5 and Kp1-specific peaks, respectively) (Figure 1 and Table 1). These data reveal the possibility to identify precisely an isolate of the *K. pneumoniae* complex based on the specific combination of the above described peaks. To the best of our knowledge, this is the first time that mass spectrometry biomarkers that discriminate all phylogroups of the *K. pneumoniae* complex are described. Furthermore, cluster analysis grouped all strains according to their phylogroup (Supplementary Figure S1), demonstrating the potential of whole spectrum comparison for strain identification at the phylogroup level.

**Table 1:** MALDI-TOF mass spectrometry peak biomarkers useful to discriminate *Klebsiella pneumoniae* phylogroups.

Present in Kp phylogroup(s)	Peak position (*m/z*)^1^	Sensitivity^2^ [95% CI]	Specificity^3^ [95% CI]	Possible proteins^4^
Kp1	4153	10/10, 100% [69.2–100.0%]	35/36, 97.2% [85.5–99.9%]	YjbJ (*double charged ion*)
	8305	10/10, 100% [69.2–100.0%]	36/36, 100% [90.3–100.0%]	YjbJ
Kp2	4136	9/9, 100% [66.4–100.0%]	37/37, 100% [90.5–100.0%]	YjbJ (*double charged ion*)
	8271	9/9, 100% [66.4–100.0%]	37/37, 100% [90.5–100.0%]	YjbJ
Kp3 and Kp5	7768	15/15, 100% [78.2–100.0%]	31/31, 100% [88.8–100.0%]	Ribosomal protein L31
Kp4	3835	7/7, 100% [59.0–100.0%]	39/39, 100% [91.0–100.0%]	YdgH (chain, *double charged ion*)
	7670	7/7, 100% [59.0–100.0%]	39/39, 100% [91.0–100.0%]	YdgH (chain)
Kp5	4777	6/6, 100% [54.1–100.0%]	40/40, 100% [91.2–100.0%]	Ribosomal protein S20 (demethionated form, *double charged ion*)
	9553	6/6, 100% [54.1–100.0%]	40/40, 100% [91.2–100.0%]	Ribosomal protein S20 (demethionated form)
Kp1 and Kp6	5278	12/15, 80% [51.9–95.7%]	41/41,100% [88.8–100.0%]	Ribosomal protein S22

*CI, confidence interval. ^*1*^Position in the spectra using a tolerance of ±0.03%. ^*2*^Proportion of true positives that are correctly identified as such. ^*3*^Proportion of true negatives that are correctly identified as such. ^*4*^As determined using TagIdent (https://web.expasy.org/tagident/tagident.html)*.

About half of the peaks visualized in a bacterial spectrum in the *m/z* range used in this work (2,000–20,000) correspond to ribosomal proteins (van Belkum et al., 2017). Here, we were able to presumptively identify four of the specific peaks as ribosomal proteins (S20, S22, and L31, respectively 4777/9553, 5278 and 7768 *m/z*, specific for Kp5, Kp1+Kp6, and Kp3+Kp5). In fact, the 5278 m/z – Kp1/Kp6 specific peak presumptively identified as S22 ribosomal protein was observed in Kp2, Kp3 and Kp5 at 5250 *m/z* and in Kp4 at 5190 *m/z* (not specific), corroborating the information obtained in the protein alignment (Supplementary Figure S2). Regarding the 7768 *m/z* Kp3/Kp5 specific peak assumed as L31 ribosomal protein, it was found in the remaining phylogroups at 7738 *m/z*, whereas the demethionated form of S20 was observed at 4769/9536 *m/z*. Furthermore, we noticed that S22 was missing in three isolates of Kp1 or Kp6, which may be explained by the fact that S22 is a stationary phase-induced protein (Yutin et al., 2012).

Regarding the specific peak pairs of Kp1, Kp2 and Kp4, we presumptively identified the Kp1 and Kp2 specific markers as YjbJ, a putative stress response protein, the sequence of which differs between the two groups and the remaining phylogroups (present at 4107/8213 *m/z*). Kp4 specific peaks were identified as the mature form of the YdgH protein (DUF1471 domain-containing protein), a periplasmatic protein involved in pathogenesis, observed in the spectra of the other phylogroups at 3851/7702 *m/z* instead of 3835/7670 *m/z* (Table 1 and Supplementary Figure S2).

The specificity of the peaks was supported by the protein alignments obtained from whole-genome sequences (Supplementary Figure S2). Interestingly, the 4153 *m/z* Kp1 specific peak was also observed with low intensity in the three replicates of the SB11-Kp2 isolate (Figure 1). However, sequence analysis of YjbJ protein (locus tag – KQQSB11_50044) revealed 100% identity with the other Kp2 strains and a theoretical molecular mass of 8274 Da (4137 in the double charged ion form). Furthermore, this peak was only present at 4153 *m/z*, corroborating the hypothesis of an unspecific peak in SB11 Kp2 isolate.

In a previous work, eight specific peaks presumptively corresponding to four different proteins were described, including three ribosomal ones (L31, S15, L28) and YjbJ, for the discrimination of Kp1 from Kp3 (Dinkelacker et al., 2018). However, in our MALDI-TOF MS experiments, we were not able to detect the presumptively demethionated form of S15 (10077/5038 *m/z* – Kp1 and 10062/5031 *m/z* – Kp3) or L28 (8875/4437 *m/z* – Kp1 and 8891/4447 *m/z* – Kp3) ribosomal proteins. However, we confirmed that the sequence alignment in our genomes showed in the case of S15 (10209 Da – Kp1, Kp2, Kp4, Kp6) a one amino-acid change for Kp3 (S79A, 10192 Da) and for Kp5 (A80S, 10225 Da). In the case of L28, one amino-acid change distinguished Kp1/Kp2/Kp4 (9007 Da) from Kp3/Kp5/Kp4 (A69S, 9022 Da), thus not being specific for any phylogroup, although we were not able to detect it in our spectra.

Using the cross-validation procedure for SVM classifier, a 98.1% rate of correct predictions (average of 10 experiments; range: 96.9–100%) for the phylogroups was found, showing that this approach is promising for identification of *K. pneumoniae* complex members. Further, an independent validation dataset of 49 isolates identified at phylogroup level by whole genome sequence analysis was used. Bacteria were grown in LB or blood agar and either protein-extracted or directly analyzed. Consistent with results obtained for the isolates of the model, the MALDI Biotyper Compass database identified the isolates either as *K. pneumoniae* (39 strains, all the Kp1, Kp2, and Kp4 isolates) or as *K. variicola* (10 strains, all Kp3 and Kp5 isolates) with identification scores ranging between 1.82 and 2.55 (mean range of 2.34). In contrast, the projection of the validation dataset spectra in our model using the SVM classifier showed that all isolates were correctly identified at the phylogroup level with high confidence. This complete identification was obtained in all experimental conditions. The results of this external validation demonstrate the potential of MALDI-TOF MS as a precise *K. pneumoniae* phylogroup identification method. Moreover, they show that the culture media used as well as the two different sample preparation procedures do not seem to affect the identification results. The use of the direct transfer procedure from blood agar, the condition most frequently used in routine laboratory conditions, thus appears appropriate for *K. pneumoniae* identification by MALDI-TOF MS.

## Conclusion

This work demonstrates the existence of *K. pneumoniae* phylogroup-specific protein biomarkers that can be detected by MALDI-TOF MS. This finding opens the possibility for industrial, veterinary or medical microbiology laboratories, to identify isolates of the *K. pneumoniae* complex at the species or phylogroup level. We urge that reference spectra of the various taxa of the *K. pneumoniae* complex be incorporated into reference MALDI-TOF spectra databases. Improved identification of *K. pneumoniae* and related taxa will advance our understanding of the epidemiology, ecology, and links with pathogenesis of this increasingly important group of pathogens.

## Author contributions

CR performed the experimental work related to the acquisition of mass spectra in MALDI-TOF MS, performed the bioinformatics analysis, and wrote the manuscript. VP and AR performed the experimental work related to sample processing, WGS and MALDI-TOF MS. SB coordinated the design of the study and methodological approach, the analysis of data, and the revision of the manuscript. All authors read and approved the final version of this manuscript.

## Conflict of Interest Statement

The authors declare that the research was conducted in the absence of any commercial or financial relationships that could be construed as a potential conflict of interest.

## Footnotes

1 https://www.bruker.com/fileadmin/user_upload/1-Products/Separations_ MassSpectrometry/MALDI_Biotyper/US_CA_System/MBT_list_of_organisms_ 10_2017.pdf

2 http://web.expasy.org/tagident/

## References

[B1] Becker L. Fuchs S. Pfeifer Y. Semmler T. Eckmanns T. Korr G. (2018). Whole genome sequence analysis of CTX-M-15 producing klebsiella isolates allowed dissecting a polyclonal outbreak scenario. *Front. Microbiol.* 9:322. 10.3389/fmicb.2018.00322 29527200 PMC5829066

[B2] Berry G. J. Loeffelholz M. J. Williams-Bouyer N. (2015). An Investigation into laboratory misidentification of a bloodstream klebsiella variicola infection. *J. Clin. Microbiol.* 53 2793–2794. 10.1128/JCM.00841-15 26063851 PMC4508421

[B3] Bialek-Davenet S. Criscuolo A. Ailloud F. Passet V. Jones L. Delannoy-Vieillard A. S. (2014a). Genomic definition of hypervirulent and multidrug-resistant *Klebsiella pneumoniae* clonal groups. *Emerg. Infect. Dis.* 20 1812–1820. 10.3201/eid2011.140206 25341126 PMC4214299

[B4] Bialek-Davenet S. Criscuolo A. Ailloud F. Passet V. Nicolas-Chanoine M. H. Decré D. (2014b). Development of a multiplex PCR assay for identification of *Klebsiella pneumoniae* hypervirulent clones of capsular serotype K2. *J. Med. Microbiol.* 63 1608–1614. 10.1099/jmm.0.081448-0 25261063

[B5] Blin C. Passet V. Touchon M. Rocha E. P. C. Brisse S. (2017). Metabolic diversity of the emerging pathogenic lineages of *Klebsiella pneumoniae*. *Environ. Microbiol.* 19 1881–1898. 10.1111/1462-2920.13689 28181409

[B6] Breurec S. Melot B. Hoen B. Passet V. Schepers K. Bastian S. (2016). Liver abscess caused by infection with community-acquired *Klebsiella quasipneumoniae* subsp. *quasipneumoniae*. *Emerg. Infect. Dis.* 22 529–531. 10.3201/eid2203.151466 26890371 PMC4766917

[B7] Brisse S. Passet V. Grimont P. A. D. (2014). Description of Klebsiella quasipneumoniae sp. nov., a novel species isolated from human infections, with two subspecies *Klebsiella quasipneumoniae* subsp. *quasipneumoniae* subsp. nov. and *Klebsiella quasipneumoniae* subsp. *similipneumoniae* subsp. nov., and a. *Int. J. Syst. Evol. Microbiol.* 64 3146–3152. 10.1099/ijs.0.062737-0 24958762

[B8] Brisse S. van Himbergen T. Kusters K. Verhoef J. (2004). Development of a rapid identification method for *Klebsiella pneumoniae* phylogenetic groups and analysis of 420 clinical isolates. *Clin. Microbiol. Infect.* 10 942–945. 10.1111/j.1469-0691.2004.00973.x 15373895

[B9] Brisse S. Verhoef J. (2001). Phylogenetic diversity of *Klebsiella pneumoniae* and *Klebsiella oxytoca* clinical isolates revealed by randomly amplified polymorphic DNA, gyrA and parC genes sequencing and automated ribotyping. *Int. J. Syst. Evol. Microbiol.* 51 915–924. 10.1099/00207713-51-3-915 11411715

[B10] DeMarco M. L. Ford B. A. (2013). Beyond identification: emerging and future uses for maldi-tof mass spectrometry in the clinical microbiology laboratory. *Clin. Lab. Med.* 33 611–628. 10.1016/j.cll.2013.03.013 23931841

[B11] Dinkelacker A. G. Vogt S. Oberhettinger P. Mauder N. Rau J. Kostrzewa M. (2018). Typing and species identification of clinical Klebsiella isolates by Fourier-transform infrared (FTIR) spectroscopy and matrix-assisted laser desorption/ionization time-of-flight (MALDI-TOF) mass spectrometry. *J. Clin. Microbiol.* 56:e00843-18. 10.1128/JCM.00843-18 30135233 PMC6204683

[B12] European Centre for Disease Prevention and Control [ECDC] (2017). “Surveillance of antimicrobial resistance in Europe 2016” in *Proceedings of the Annual report of the European Antimicrobial REsistance Surveillance Network (EARS-Net)*, (Solna Municipality: European Centre for Disease Prevention and Control), 10.2900/296939

[B13] Fagerquist C. K. (2017). Unlocking the proteomic information encoded in MALDI-TOF-MS data used for microbial identification and characterization. *Expert Rev. Proteomics* 14 97–107. 10.1080/14789450.2017.1260451 27838927

[B14] Fonseca E. L. Ramos N. D. Andrade B. G. Morais L. L. Marin M. F. Vicente A. C. (2017). A one-step multiplex PCR to identify *Klebsiella pneumoniae, Klebsiella variicola*, and *Klebsiella quasipneumoniae* in the clinical routine. *Diagn. Microbiol. Infect. Dis.* 87 315–317. 10.1016/j.diagmicrobio.2017.01.005 28139276

[B15] Garza-Ramos U. Silva-Sánchez J. Martínez-Romero E. Tinoco P. Pina-Gonzales M. Barrios H. (2015). Development of a Multiplex-PCR probe system for the proper identification of *Klebsiella variicola*. *BMC Microbiol.* 15:64. 10.1186/s12866-015-0396-6 25886267 PMC4361152

[B16] Haeggman S. Lofdahl S. Paauw A. Verhoef J. Brisse S. (2004). Diversity and evolution of the class a chromosomal beta-lactamase gene in *Klebsiella pneumoniae*. *Antimicrob. Agents Chemother.* 48 2400–2408. 10.1128/AAC.48.7.2400-2408.2004 15215087 PMC434173

[B17] Holt K. E. Wertheim H. Zadoks R. N. Baker S. Whitehouse C. A. Dance D. (2015). Genomic analysis of diversity, population structure, virulence, and antimicrobial resistance in *Klebsiella pneumoniae*, an urgent threat to public health. *Proc. Natl. Acad. Sci. U.S.A.* 112 E3574–E3581. 10.1073/pnas.1501049112 26100894 PMC4500264

[B18] Long S. W. Linson S. E. Ojeda Saavedra M. Cantu C. Davis J. J. Brettin T. (2017a). Whole-genome sequencing of a human clinical isolate of the novel species *Klebsiella quasivariicola* sp. nov. *Genome Announc.* 5:e01057-17. 10.1128/genomeA.01057-17 29051239 PMC5646392

[B19] Long S. W. Linson S. E. Ojeda Saavedra M. Cantu C. Davis J. J. Brettin T. (2017b). Whole-genome sequencing of human clinical *Klebsiella pneumoniae* isolates reveals misidentification and misunderstandings of *Klebsiella pneumoniae, Klebsiella variicola*, and *Klebsiella quasipneumoniae*. *mSphere* 2:e00290-17. 10.1128/mSphereDirect.00290-17 28776045 PMC5541162

[B20] Maatallah M. Vading M. Kabir M. H. Bakhrouf A. Kalin M. Nauclér P. (2014). *Klebsiella variicola* is a frequent cause of bloodstream infection in the Stockholm area, and associated with higher mortality compared to *K. pneumoniae*. *PLoS One* 9:113539. 10.1371/journal.pone.0113539 25426853 PMC4245126

[B21] Passet V. Brisse S. (2018). Description of *Klebsiella grimontii* sp. nov. *Int. J. Syst. Evol. Microbiol.* 68 377–381. 10.1099/ijsem.0.002517 29205126

[B22] Rodrigues C. Passet V. Brisse S. (2018). Identification of *Klebsiella pneumoniae* complex members using MALDI-TOF mass spectrometry. *bioRxiv* [Preprint]. 10.1101/350579 PMC6294014 30581423

[B23] Rosenblueth M. Martínez L. Silva J. Martínez-Romero E. (2004). *Klebsiella variicola*, a novel species with clinical and plant-associated isolates. *Syst. Appl. Microbiol.* 27 27–35. 10.1078/0723-2020-00261 15053318

[B24] Seki M. Gotoh K. Nakamura S. Akeda Y. Yoshii T. Miyaguchi S. (2013). Fatal sepsis caused by an unusual Klebsiella species that was misidentified by an automated identification system. *J. Med. Microbiol.* 62 801–803. 10.1099/jmm.0.051334-0 23449877

[B25] van Belkum A. Welker M. Pincus D. Charrier J. P. Girard V. (2017). Matrix-assisted laser desorption ionization time-of-flight mass spectrometry in clinical microbiology: what are the current issues? *Ann. Lab. Med.* 37 475–483. 10.3343/alm.2017.37.6.475 28840984 PMC5587819

[B26] Vranckx K. De Bruyne K. Pot B. (2017). “Analysis of MALDI-TOF MS spectra using the bionumerics software,” in *MALDI-TOF Tandem MS Clinical Microbiology*, eds Shah H. N. Gharbia S. E. (Hoboken, NJ: John Wiley & Sons), 539–562. 10.1002/9781118960226.ch21

[B27] Wyres K. L. Holt K. E. (2016). *Klebsiella pneumoniae* population genomics and antimicrobial-resistant clones. *Trends Microbiol.* 24 944–956. 10.1016/j.tim.2016.09.007 27742466

[B28] Yutin N. Puigbò P. Koonin E. V. Wolf Y. I. (2012). Phylogenomics of prokaryotic ribosomal proteins. *PLoS One* 7:e36972. 10.1371/journal.pone.0036972 22615861 PMC3353972

