# Supplementary material for: Identification of Klebsiella pneumoniae, Klebsiella quasipneumoniae, Klebsiella variicola and Related Phylogroups by MALDI-TOF Mass Spectrometry
Source: Front Microbiol. 2018 Dec 7;9:3000. doi: 10.3389/fmicb.2018.03000 (PMC6294014; doi:10.3389/fmicb.2018.03000)
Supplement: Supplementary file 1 [file Data_Sheet_1.docx]

Supplementary Material

Identification of *Klebsiella pneumoniae, Klebsiella quasipneumoniae, Klebsiella variicola* and related phylogroups by MALDI-TOF mass spectrometry

Carla Rodrigues, Virginie Passet, Andriniaina Rakotondrasoa, Sylvain Brisse^*^

*** Correspondence:** Sylvain Brisse: sylvain.brisse@pasteur.fr

# Table S1. Strains included in the study.

| **Taxonomic Designation** | **Strain bank ID^1^** | **Strain Name** | **PhG** | **MLST** | **Isolation Year** | **Source** | **Host** | **Continent** | **Country** | **Acession Number** |
| --- | --- | --- | --- | --- | --- | --- | --- | --- | --- | --- |
| *K. pneumoniae* | **SB1067** | SB4-2 | **Kp1** | 17 | 2002 | Feces | Human carriage | Europe | Netherlands | ERS2786693 |
| *K. pneumoniae* | **SB132** | ATCC 13883^T^ (DSM 30104^T^) | **Kp1** | 3 | n.a. | Blood | Human | n.a. | n.a. | GCA_000742135 |
| *K. pneumoniae* | **SB107** | MGH 78578 | **Kp1** | 38 | 1994 | Blood | Human | n.a. | n.a. | CP000647 |
| *K. pneumoniae* | **SB1139** | SB1139 | **Kp1** | 37 | 2002 | Feces | Human carriage | Europe | Netherlands | ERS2786694 |
| *K. pneumoniae* | **SB617** | 5-2 | **Kp1** | 55 | 2000 | Natuurgebied canal | Water | Europe | Netherlands | ERS2786695 |
| *K. pneumoniae* | **SB20** | 04A025 | **Kp1** | 15 | 1997 | Blood | Human | Europe | France | ERS2786696 |
| *K. pneumoniae* | **SB612** | 2-3 | **Kp1** | 50 | 2000 | Rijnhauwen bridge | Water | Europe | Netherlands | ERS2786697 |
| *K. pneumoniae* | **SB4938** | Kp13 | **Kp1** | 442 | 2009 | Blood | Human | America | Brazil | CP003999 |
| *K. pneumoniae* | **SB3928** | NTUH-K2044 | **Kp1** | 23 | n.a | Blood | Human | Asia | Taiwan | AP006725 |
| *K. pneumoniae* | **SB4496** | BJ1-GA | **Kp1** | 380 | 2011 | Liver abscess | Human | Europe | France | ERS2786698 |
| *K. quasipneumoniae* subsp. *quasipneumoniae* | **SB11** | 01A030^T^ | **Kp2** | 1528 | 1997 | Blood | Human | Europe | Austria | GCA_000751755 |
| *K. quasipneumoniae* subsp. *quasipneumoniae* | **SB1124** | SB1124 | **Kp2** | 2274 | 2002 | Canal water | Environment | Europe | Netherlands | ERS2786699 |
| *K. quasipneumoniae* subsp. *quasipneumoniae* | **SB2110** | U41 | **Kp2** | 526 | 1990 | Environment | Environment | Europe | Germany | ERS2786700 |
| *K. quasipneumoniae* subsp. *quasipneumoniae* | **SB224** | 10A442 | **Kp2** | n.d. | 1998 | Blood | Human | Europe | Italy | n.a. |
| *K. quasipneumoniae* subsp. *quasipneumoniae* | **SB2478** | 99-1002 | **Kp2** | *rpoB*18 | 1999 | n.a. | Human | Europe | Netherlands | n.a. |
| *K. quasipneumoniae* subsp. *quasipneumoniae* | **SB255** | 18A451 | **Kp2** | *gapA*17 *rpoB*18 | 1998 | Blood | Human | Europe | Spain | n.a. |
| *K. quasipneumoniae* subsp. *quasipneumoniae* | **SB3445** | 11128 | **Kp2** | 144 | n.a. | Diarrhoea | Human | n.a. | n.a. | n.a. |
| *K. quasipneumoniae* subsp. *quasipneumoniae* | **SB59** | 18A69 | **Kp2** | 1118 | 1997 | Blood | Human | Europe | Spain | ERS2786701 |
| *K. quasipneumoniae* subsp. *quasipneumoniae* | **SB98** | Kleb Ali 0320584 | **Kp2** | 622 | n.a. | Environment | Environment | n.a. | n.a. | ERS2786702 |
| *K. variicola* | **SB1** | 01A065 | **Kp3** | 2273 | 1997 | Blood | Human | Europe | Austria | ERS2786703 |
| *K. variicola* | **SB31** | 07A058 | **Kp3** | *gapA*16 *rpoB*22 | 1997 | Blood | Human | Europe | Germany | n.a. |
| *K. variicola* | **SB3278** | IPEUC-1516 | **Kp3** | *gapA*16 *infB*18 *mdh*21 *pgi*2*7 rpo*B17 | 1988 | Urine | Human | Europe | France | n.a. |
| *K. variicola* | **SB3295** | CIP 53.24 | **Kp3** | 207 | n.a. | n.a. | n.a. | n.a. | n.a. | ERS2787321 |
| *K. variicola* | **SB3301** | CIP 53.26, (1756/51) | **Kp3** | 1218 | n.a. | n.a. | n.a. | n.a. | n.a. | ERS2787322 |
| *K. variicola* | **SB48** | F2R9^T^ | **Kp3** | 2263 | n.a. | Banana | Food | America | Mexico | CP010523 |
| *K. variicola* | **SB489** | 6115; KLSP49 | **Kp3** | *rpoB*22 | n.a. | n.a. | n.a. | n.a. | n.a. | n.a. |
| *K. variicola* | **SB497** | 4425/51 | **Kp3** | 1217 | n.a. | n.a. | n.a. | n.a. | n.a. | ERS2787323 |
| *K. variicola* | **SB579** | Kp342 | **Kp3** | 146 | n.a. | Maize | Plant | America | USA | CP000964 |
| K. *quasipneumoniae* subsp. *similipneumoniae* | **SB164** | CRBIP28.12,  (09A323) | **Kp4** | 18 | 1997 | Blood | Human | Europe | Greece | ERS2786705 |
| K. *quasipneumoniae* subsp. *similipneumoniae* | **SB203** | 12A476 | **Kp4** | 384 | 1998 | Blood | Human | Europe | Netherlands | ERS2786706 |
| K. *quasipneumoniae* subsp. *similipneumoniae* | **SB30** | 07A044^T^ | **Kp4** | 1215 | 1997 | Blood | Human | Europe | Germany | GCA_000613225 |
| K. *quasipneumoniae* subsp. *similipneumoniae* | **SB3233** | 325 | **Kp4** | 488 | 1975 | n.a. | Human | Europe | France | n.a. |
| K. *quasipneumoniae* subsp. *similipneumoniae* | **SB3297** | CIP 52.200, (1303/50) | **Kp4** | 1224 | n.a. | n.a. | n.a. | n.a. | Turkey | ERS2787324 |
| K. *quasipneumoniae* subsp. *similipneumoniae* | **SB4697** | CIP 110288  (CW-D3) | **Kp4** | 2275 | 2010 | Farmland soil | Environment | Asia | China | ERS2786707 |
| K. *quasipneumoniae* subsp. *similipneumoniae* | **SB610** | 1-1 | **Kp4** | 2276 | 2000 | Lake kikker | Environment | Europe | Netherlands | ERS2786708 |
| not defined | **SB5387** | 814 | **Kp5** | 2466 | 2015 | Fecal sample | Human | Africa | Madagascar | ERS2787526 |
| not defined | **SB5531** | 1266 | **Kp5** | 2561 | 2016 | Fecal sample | Human | Africa | Madagascar | ERS2787528 |
| not defined | **SB5544** | 1283 | **Kp5** | 2605 | 2016 | Fecal sample | Human | Africa | Madagascar | ERS2787529 |
| not defined | **SB5610** | 1375 | **Kp5** | 2600 | 2016 | Fecal sample | Human | Africa | Madagascar | ERS2787530 |
| not defined | **SB824** | Gal12 | **Kp5** | *gapA*181 *rpoB*189 | n.a. | Environment | Coffee plant | America | Mexico | n.a. |
| not defined | **SB94** | CDC 4241-71 | **Kp5** | 1216 | n.a. | Environment | Environment | n.a. | n.a. | ERS2786710 |
| not defined  (*K. quasivariicola*) | **SB33** | 08A119 | **Kp6** | 1214 | 1997 | Blood | Human | Europe | Germany | ERS2786709 |
| not defined  (*K. quasivariicola*) | **SB6071** | 10982 | **Kp6** | 1155 | 2005 | Peri-rectal | Human | America | USA | GCA_000523395 |
| not defined  (*K. quasivariicola*) | **SB6096** | KPN1705 | **Kp6** | 209 | 2014 | Wound | Human | America | USA | CP022823 |
| not defined  (*K. quasivariicola*) | **SB6094** | 01-467-2ECBU | **Kp6** | 2830 | 2015 | Feces | Human | Africa | Madagascar | ERS2787531 |
| not defined  *(K. quasivariicola*) | **SB6095** | 01-310A | **Kp6** | 3303 | 2013 | Vaginal swab | Human | Africa | Madagascar | ERS2787532 |

^1^Internal strain collection number of the Biodiversity and Epidemiology of Bacterial Pathogens unit, Institut Pasteur.

^T^ , Type strain

PhG, Phylogroup; n.a., not available; n.d., not determined

**Supplementary Figure S1.** Neighbor Joining tree based on ranked Pearson correlation constructed with MALDI-TOF MS spectra of 46 *Klebsiella pneumoniae* complex strains.

##

Each node represents the spectrum of a *K. pneumoniae* complex strain, and the different phylogroups (previously determined by WGS or sequencing of a specific genetic marker) are shown in different colors. Note that the strains were distributed into branches according to their phylogroup.

**Supplementary Figure S2.** Amino acid sequence alignments and the respective molecular weight of the presumptive proteins associated with specific MALDI-TOF MS peaks in *K. pneumoniae* phylogroups.

Amino acid substitutions leading to mass changes are represented in bold.

Grey labels represent the signal peptide of the protein, usually removed in the mature protein (chain).

Mw, molecular weight.
